# Diaphragmatic Ultrasound in Neonates with Transient Tachypnea: Comparison with Healthy Controls and Inter-Operator Reliability

**DOI:** 10.3390/children13010024

**Published:** 2025-12-23

**Authors:** Maria Letizia Patti, Carmela Crapanzano, Rosa Maria Cerbo, Federico Schena, Anna La Rocca, Valeria Cortesi, Giacomo Simeone Amelio, Stefano Ghirardello

**Affiliations:** 1 Fondazione IRCCS Policlinico San Matteo, 27100 Pavia, Italy; c.crapanzano@smatteo.pv.it (C.C.); rm.cerbo@smatteo.pv.it (R.M.C.); f.schena@smatteo.pv.it (F.S.); v.cortesi@smatteo.pv.it (V.C.); g.amelio@smatteo.pv.it (G.S.A.); s.ghirardello@smatteo.pv.it (S.G.); 2 Cardinal Massaia Hospital, 14100 Asti, Italy; alarocca@asl.at.it

**Keywords:** transient tachypnea, diaphragmatic function, lung ultrasound, diaphragmatic excursion, diaphragmatic thickness

## Abstract

**Highlights:**

**What are the main findings?**

• Diaphragmatic excursion increases during the first 48 h in healthy neonates.
• On day two, TTN infants show lower diaphragmatic excursion compared with controls, and a negative correlation develops between excursion and LUS, indicating impaired diaphragmatic function in the context of lung disease.

**What are the implications of the main findings?**

• Diaphragmatic ultrasound may help identify early functional impairment in neonates with TTN, complementing lung ultrasound to characterize disease severity.
• Integrated lung–diaphragm ultrasound assessment may support monitoring of disease progression and guide decisions on respiratory support, especially during the first 48 h of life.

**Abstract:**

**Background:** The role of diaphragmatic function in transient tachypnea of the newborn (TTN) remains poorly understood. This study aimed to compare diaphragmatic ultrasound parameters between neonates with TTN requiring non-invasive ventilation (NIV) and healthy neonates. Secondary objectives include the relationships between these parameters and gestational age (GA), birth weight (BW), and the evaluation of inter-operator reproducibility. **Methods:** This prospective observational pilot study involved neonates with GA ≥ 34 weeks with clinical and ultrasound diagnosis of TTN treated with NIV. An equal number of healthy neonates served as controls. Diaphragmatic and lung ultrasound were performed on day 1 (T0) and day 2 (T1) of life. Measurements included end-inspiratory and end-expiratory diaphragmatic thickness (DTi and DTe, respectively), diaphragmatic excursion (DE), and Lung Ultrasound Score (LUS). Inter-operator reproducibility was tested in 31 neonates (62 scans in total). **Results:** Forty neonates were enrolled (20 TTN, 20 controls). DE was significantly higher in controls compared with TTN neonates (4.6 ± 0.9 mm vs. 5.4 ± 1.3 mm, *p* = 0.03) and increased from T0 to T1 in the control group (4.6 ± 1.1 mm vs. 5.4 ± 1.3 mm, *p* = 0.04), while no significant variation was observed in TTN cases. A negative correlation, approaching significance, was found between DE and LUS at T1 (*p* = 0.05). DTi and DTe increased linearly with GA and BW (*p* < 0.001). Bland–Altman analysis showed low bias and acceptable limits of agreement between measurements. **Conclusions:** The underlying pulmonary disease may influence diaphragmatic function in neonates with TTN. The integration of lung and diaphragmatic ultrasound could be useful for monitoring disease progression and follow-up.

## 1. Introduction

Recent advances in neonatal imaging have emphasized the importance of diaphragmatic function in respiratory adaptation after birth. Ultrasound assessment of the diaphragm has become a promising, non-invasive method to evaluate diaphragmatic performance in neonates, allowing measurement of parameters such as diaphragmatic thickness, thickening fraction, excursion, and fractional shortening [1,2,3]. Studies in neonates needing mechanical ventilation have shown significant diaphragmatic atrophy and decreased thickening fractions during ventilation [4,5]. Transient tachypnea of the newborn (TTN) remains one of the most common causes of neonatal respiratory distress, especially in late preterm and term infants. It is mainly caused by delayed clearance of fetal lung fluid, leading to impaired gas exchange and increased work of breathing. Although TTN is usually self-limited, resolving within 24–72 h, it can still require respiratory support and may affect respiratory outcomes in early childhood [6,7]. The main goal of this study was to compare diaphragmatic functional parameters (end-inspiratory and end-expiratory thickness, thickening fraction, and diaphragmatic excursion) between neonates requiring non-invasive ventilation (NIV) and healthy term neonates. Additional objectives included assessing how these parameters vary with gestational age and birth weight, and evaluating the reproducibility of diaphragmatic ultrasound measurements.

## 2. Materials and Methods

This prospective observational pilot case–control study was conducted at the Neonatology and Neonatal Intensive Care Unit of IRCCS Policlinico San Matteo, Pavia, between February and July 2024. All inborn neonates with a gestational age of ≥34 weeks were considered eligible. Infants who developed respiratory distress after stabilization in the delivery room, requiring non-invasive respiratory support (nasal Continuous Positive Airway Pressure [nCPAP] or Heated Humidified High-Flow Nasal Cannula [HHHFNC]), and with a clinical and ultrasonographic diagnosis of transient tachypnea of the newborn (TTN), were included in the case group. An equal number of healthy neonates with gestational age ≥34 weeks, admitted to the Neonatology or Rooming-in unit, were selected as controls. Parental written informed consent was obtained before enrolment in both groups. Newborns with gestational age below 34 weeks, respiratory distress syndrome (RDS), hemodynamic instability, major congenital malformations, surgical conditions, or known metabolic/genetic syndromes were excluded. TTN was clinically defined as the presence of tachypnoea (respiratory rate > 60 breaths/min) and signs of respiratory distress (including thoracic and abdominal movement, intercostal and xiphoid retraction, nasal flaring, and expiratory grunt, Silverman score > 1) occurring within the first 24 h of life. The diagnosis of TTN was confirmed, as per usual clinical practice in our department, by lung ultrasound, showing a thickened pleural line, multiple B-lines alternating with A-lines, and the presence of the characteristic “double lung point” sign [8]. For both groups, the following clinical data were collected and recorded in a dedicated database: gestational age, gender, mode of delivery, maternal steroid prophylaxis, anthropometric parameters at birth (weight, length, head circumference), parity, Apgar scores at 1 and 5 min, and need for resuscitation at birth. For the TTN group, additional data were collected at each ultrasound assessment, including the mode and duration of non-invasive ventilation, Silverman score, and respiratory rate at the time of examination.

### 2.1. Ultrasound Equipment and Technique

Both diaphragmatic and lung ultrasound examinations were performed using a Hitachi Aloka Arietta V70™ (Hitachi Aloka Medical America, Inc. 10 Fairfield Boulevard, Wallingford, CT, USA) ultrasound system by two physicians with experience in pulmonary ultrasound, during routine clinical care. All images were reanalyzed for the purpose of the study by a senior neonatologist with expertise in LUS. Diaphragmatic ultrasound measurements included end-inspiratory and end-expiratory thickness (DTi and DTe, respectively), diaphragmatic thickening fraction (DTF), and diaphragmatic excursion (DE), while lung ultrasound was utilized to assess the Lung Ultrasound Score (LUS) [9].

In both groups, ultrasound evaluations were performed by either one of the two operators, at two different time points: T0 (within 6 h after birth) and T1 (second day of life, between 24 h and 36 h after birth), with the neonate in the supine position, during quiet sleep or calm wakefulness.

Diaphragmatic ultrasound was performed using a high-frequency linear transducer (15–8 MHz) positioned perpendicularly to the chest wall between the 8th and 10th intercostal spaces, along the right anterior to mid-axillary line. In B-mode, the diaphragm was identified as a hypoechoic structure bordered by two echogenic lines corresponding to the pleura (superiorly) and the peritoneum (inferiorly).

**Figure 1:**
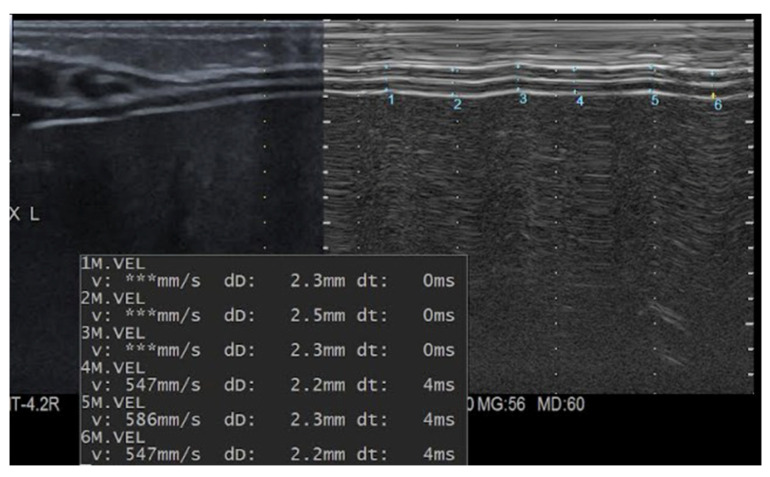
Measurement of DTi and Dte in M-Mode scan. Measurement n.1, 3, 5 indicates DTi; measurement n.2, 4, 6 indicates DTe. DTi Diaphragmatic Inspiratory thickness; DTe Diaphragmatic Expiratory thickness.

End-inspiratory and end-expiratory diaphragm thicknesses were measured in M-mode by calculating the maximal and minimal distance between the pleural and peritoneal layers (Figure 1). To ensure measurement accuracy and reproducibility, three measurements were obtained over three consecutive respiratory cycles, and the mean value was used for analysis.

The diaphragmatic thickening fraction (DTF) was calculated using the following formula:

$$\mathrm{DTF}=\frac{\mathrm{End}\text{-}\mathrm{inspiratory}\;\mathrm{thickness}-\mathrm{End}\text{-}\mathrm{expiratory}\;\mathrm{thickness}}{\mathrm{End}\text{-}\mathrm{expiratory}\;\mathrm{thickness}}\times 100\%$$

**Figure 2:**
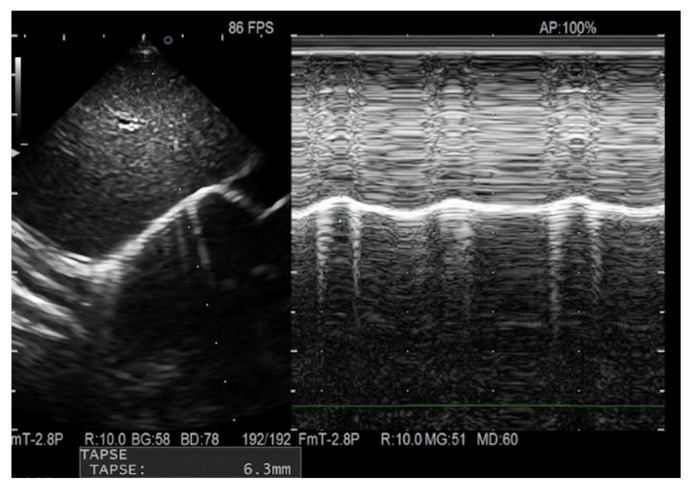
Measurement of DE in M-Mode scan. DE, Diaphragmatic excursion.

Diaphragmatic excursion was assessed in M-mode using a low-frequency phased-array transducer (14–3 MHz) placed in the subcostal area between the right midclavicular and anterior axillary lines, with the confluence of the hepatic veins as an anatomical landmark. Excursion was measured as the distance between the baseline at end-expiration and the point of maximal inspiration (Figure 2).

Simultaneously, lung ultrasound was performed using a high-frequency linear transducer (15–8 MHz). The LUS score was assigned by evaluating six lung regions: upper anterior, lower anterior, and lateral regions on both sides of the chest. In detail, the LUS score was assigned as follows, according to Brat R. et al. [9]: 0 indicates an A-pattern, characterized by the exclusive presence of A-lines; 1 corresponds to a B-pattern, defined by the appearance of three or more well-separated B-lines; 2 refers to a severe B-pattern, in which B-lines are densely packed and merging, possibly accompanied by small subpleural consolidations; 3 is used when larger, more extensive consolidations are present. For each lung area, a 0- to 3-point score was given (total score ranging from 0–18).

### 2.2. Assessment of Reproducibility

To assess the reproducibility of the diaphragmatic ultrasound, measurements were independently carried out by two different operators (C.C. and A.L.R.) on a subgroup of 31 neonates (independently cases or controls), with a total of 62 scans and a 5-min gap between assessments.

### 2.3. Statistical Analysis

Statistical analysis was performed using DATAtab software (DATAtab e.U., Graz, Austria). The Shapiro–Wilk test was used to assess the normality of data distribution [10] (Supplementary Table S1). Variables with normal distribution (*p* > 0.05) were analyzed using parametric tests. Homogeneity of variances was assessed using Levene’s test [11]. When the assumption of equal variances was violated (*p* < 0.05), Welch’s correction was applied [12,13]. The independent-sample Student’s t-test was applied to compare continuous parametric variables between groups. In contrast, the paired-samples t-test was used to compare numerical variables within the same group. The Mann–Whitney U test was applied for non-parametric continuous variables. Categorical variables were compared using the Chi-square test or Fisher’s exact test, as appropriate. Clinical and ultrasound data were expressed as mean ± standard deviation (SD) or median (interquartile range, IQR), depending on distribution. Categorical variables were presented as absolute numbers and percentages. Pearson’s correlation coefficient was used for correlations between normally distributed variables, and Spearman’s rank correlation coefficient for non-normally distributed variables. A point-biserial correlation was used to assess the association between diaphragmatic excursion and the type of ventilation. A *p*-value < 0.05 was considered statistically significant. Given the pilot nature of the study and the limited number of predefined outcomes, no correction for multiple comparisons was applied, and effect size calculations were not included in the analysis plan. Inter-operator agreement was evaluated using Bland–Altman plots and the coefficient of variation (CV), while reliability was assessed with the intraclass correlation coefficient (ICC) from a two-way random-effects analysis of variance [14,15].

## 3. Results

Of the 65 neonates initially considered eligible, 44 were enrolled, and 40 of them (20 cases and 20 controls) completed the study protocol. Demographic and prenatal characteristics of the two groups are presented in Table 1.

Data analysis revealed no significant differences in diaphragmatic parameters between cases and controls on the first day of life (Table 2, T0). On the second day (Table 2, T1), diaphragmatic excursion was significantly greater in healthy neonates than in those with TTN (*p* = 0.03). Healthy newborns, unlike the TTN group, also exhibited a significant increase in diaphragmatic excursion from the first to the second day (*p* = 0.04) (Table 3). No correlation between diaphragmatic excursion measured on T1 and Silverman score, or type and duration of respiratory support, was found. Instead, a negative correlation between diaphragmatic excursion and LUS score was found at the threshold of statistical significance (*r* = −0.44; *p* = 0.05) (Supplement, Table S2).

Analysis of the entire cohort showed a significant correlation between diaphragmatic thicknesses, gestational age, and birth weight (*p* < 0.01) (Table 4). Bland–Altman plots showed low bias values (0.18 for end-inspiratory thickness, 0.15 for end-expiratory thickness, 0.3 for diaphragmatic excursion) and acceptable limits of agreement between ultrasound measurements performed by the two operators (95% CI: −1.19 to 0.83 for end-inspiratory thickness; −0.98 to 0.68 for end-expiratory thickness; −2.74 to 2.15 for diaphragmatic excursion) (Supplement Figures S1–S3). ICC showed good reliability for end-expiratory thickness (ICC = 0.66), moderate reliability for end-inspiratory thickness (ICC = 0.52), and discrete reliability for diaphragmatic excursion (ICC = 0.32) (Table 5).

## 4. Discussion

Diaphragmatic ultrasound has been widely used in adult and pediatric populations to assess diaphragmatic dysfunction in patients undergoing invasive mechanical ventilation, particularly in relation to the duration of ventilatory support and extubation outcomes [2]. However, studies evaluating diaphragmatic function in association with underlying lung disease remain limited, especially in neonates.

Our pilot study compared diaphragmatic parameters in healthy neonates and those with TTN requiring non-invasive ventilation during the first 48 h of life. Diaphragmatic thickness and excursion measured within the first 24 h were similar between groups, suggesting that early diaphragmatic function may not be significantly influenced by underlying pulmonary disease during the initial transition to extrauterine life.

However, diaphragmatic excursion was the only parameter that showed a significant increase from the first to the second day of life in healthy neonates. This progressive increase may be related to the gradual maturation of respiratory muscles over time. Furthermore, diaphragmatic excursion was significantly greater in healthy infants compared with those with TTN after the first 24 h of life, suggesting partial diaphragmatic impairment possibly related to reduced lung compliance due to respiratory disease.

This hypothesis is further supported by our finding of a negative correlation—at the threshold of statistical significance (*p* = 0.05)—between diaphragmatic excursion on the second day of life and the LUS score, indicating that lower excursion values are associated with higher LUS scores, which reflect increased interstitial fluid content.

In contrast, analysis of our data revealed no significant differences in diaphragmatic excursion based on the type or duration of non-invasive ventilation, likely due to the small sample size and short duration of non-invasive ventilation in our cohort, though consistent with the expected clinical course of TTN. Similarly, a recent study showed that diaphragmatic function parameters, including excursion, were not predictive of successful CPAP weaning, whereas the LUS score demonstrated good sensitivity and specificity for this purpose [16]. Moreover, El-Mogy et al. reported no significant differences in diaphragmatic parameters between infants receiving nCPAP and those on HHHFNC [17]. Conversely, Gupta et al. found that in preterm neonates, diaphragmatic excursion decreased, and thickening fraction increased, among infants who failed nCPAP transition [18].

Consistent with existing literature, diaphragmatic thickness increased proportionally with gestational age and birth weight, while this was not observed for thickening fraction or diaphragmatic excursion. Rehan et al. examined diaphragmatic ultrasound parameters in 34 preterm infants and demonstrated that diaphragmatic thickness was directly proportional to anthropometric measures (birth weight, length, and head circumference) and post-conceptional age, while diaphragmatic excursion decreased with advancing post-conceptional age due to reduced chest wall compliance [19]. Similar findings were reported in a recent study of 107 preterm neonates [20]. Likewise, El-Halaby et al. observed a positive correlation between diaphragmatic thickness and excursion and anthropometric parameters in a pediatric population aged one month to two years [3]. Our findings align with these results, demonstrating a positive correlation between gestational age, birth weight, and diaphragmatic thickness. Previous studies in neonatal [3], pediatric [21], and adult [22] populations have also shown that diaphragmatic excursion tends to increase with body weight. However, in our cohort, this relationship did not reach statistical significance.

A study involving 66 healthy neonates (33 term and 33 preterm) reported that term infants exhibited greater diaphragmatic thickness compared with preterm infants, while thickening fraction was similar between groups [23].

In line with previous studies, in our cohort, the thickening fraction did not vary with gestational age. This parameter, which reflects diaphragmatic contractility, tended to be higher in neonates with TTN compared with controls on both days of life, although the difference was not statistically significant. This trend may reflect increased respiratory effort in infants with respiratory disease, as previously described in neonates with bronchopulmonary dysplasia (BPD) [24] or may be related to altered chest wall compliance and/or increased lung stiffness associated with TTN. In adults with chronic obstructive pulmonary disease (COPD), a similar diaphragmatic excursion reduction has also been reported [25,26]. However, none of these studies involve newborns.

Assessment of inter-operator agreement demonstrated good reliability for diaphragmatic thickness measurements, despite their millimetric precision. Discrete reliability was observed for diaphragmatic excursion. Inter-operator variability for this measurement may depend on the infant’s condition at the time of examination, as excursion is influenced by respiratory rate and comfort state. Overall, our results are consistent with previous pediatric and adult studies evaluating the reproducibility of this technique [22].

We are aware that the differences in diaphragmatic thickness and diaphragmatic excursion between the two groups are minimal. However, our study highlights the potential role of diaphragmatic activity during perinatal respiratory transition and how reduced lung compliance, due to an underlying disease (such as transient tachypnea in our cohort), may affect diaphragmatic function. A study with an ad hoc design (for example, with an initial assessment of diaphragmatic excursion a few hours after birth) and a larger population would be required to confirm our findings. Moreover, as our study population included only term and late preterm infants, diaphragmatic function in other pulmonary conditions, such as respiratory distress syndrome, could not be evaluated, as these are more prevalent in infants of lower gestational age.

## 5. Conclusions

Respiratory disease may negatively affect diaphragmatic function after neonatal adaptation. In healthy neonates, diaphragmatic function, particularly excursion, improves over the first hours of life, whereas this increase does not occur in infants with TTN. Diaphragmatic ultrasound is a non-invasive, bedside-applicable, and radiation-free tool that, combined with lung ultrasound, provides clinically relevant information in neonates with respiratory distress. Integrated assessment may help monitor lung disease progression and guide respiratory support weaning. Further studies are needed to improve interpretation and support routine use in neonatal intensive care units.

## Figures and Tables

**Table 1:** Characteristics of study population. The data are presented as mean (SD), median (IQR), or numbers and percentages.

	Cases (n.20)	Controls (n.20)	*p* Value
**Gestational age (weeks), mean (±SD)**	37.2 (±2.2)	38.7 (±1.9)	0.014
**Birth weight (g), mean (±SD)**	2696.4 (±641.2)	2938.5 (±608.2)	0.22
**Birth length (cm), mean (±SD)**	46.9 (±5.8)	48.2 (±3.1)	0.39
**Birth cranial circumference (cm), mean (±SD)**	33.4 (±2.3)	33.4 (±2.3)	0.71
**Small for gestational age, n (%)**	1 (5)	4 (20)	0.34
**Male sex, n (%)**	11 (55)	6 (30)	0.20
**Single born, n (%)**	15 (75)	17 (85)	0.69
**Mode of delivery (cesarean section), n (%)**	13 (65)	10 (50)	0.52
**Antenatal steroid therapy, n (%)**	7 (35)	3 (15)	0.27
**Apgar score 1 min, median (IQR)**	8 (7–9)	9 (9–9)	0.009
**Apgar score 5 min, median (IQR)**	9 (8–9)	10 (10–10)	<0.001

**Table 2:** Comparison of diaphragmatic parameters between the two groups at each time point (T0 and T1). DTi Diaphragmatic Inspiratory thickness; DTe Diaphragmatic Expiratory thickness; DTf Diaphragm thickening fraction; DE, Diaphragmatic excursion; SD standard deviation.

		T0			T1	
	TTN (n. 20)	Controls (n. 20)	*p* Value	TTN (n. 20)	Controls (n. 20)	*p* Value
**DTi (mm), mean (±SD)**	2.4 (±0.5)	2.6 (±0.4)	0.32	2.5 (±0.7)	2.6 (±0.6)	0.60
**DTe (mm), mean (±SD)**	2.1 (±0.5)	2.3 (±0.3)	0.19	2.2 (±0.6)	2.4 (±0.6)	0.36
**DTf (%), mean (±SD)**	12.9 (±6.4)	11.9 (±6.4)	0.61	14.9 (±7.5)	12.1 (±7.3)	0.23
**DE (mm), mean (±SD)**	4.3(±0.9)	4.63 (±1.1)	0.35	4.6(±0.9)	5.4 (±1.3)	0.03

**Table 3:** Variation in diaphragmatic parameters from T0 to T1. TTN: transient tachypnea of newborn; DTi: Diaphragmatic Inspiratory thickness; DTe: Diaphragmatic Expiratory thickness; DTf: Diaphragm thickening fraction; DE: Diaphragmatic excursion; SD standard deviation.

	TTN (n. 20)			Controls (n. 20)		
	T0	T1	*p* Value	T0	T1	*p* Value
**DTi (mm), mean (±SD)**	2.4 (±0.5)	2.5 (± 0.7)	0.50	2.6 (±0.4)	2.6 (±0.6)	0.44
**DTe (mm), mean (±SD)**	2.1 (±0.5)	2.2 (±0.6)	0.59	2.3 (±0.3)	2.4 (±0.6)	0.47
**DTf (%), mean (±SD)**	12.9 (±6.41)	14.9 (±7.5)	0.39	11.9 (±6.4)	12.1 (±7.3)	0.94
**DE (mm), mean (±SD)**	4.3 (±0.9)	4.6 (±0.9)	0.36	4.6 (±1.1)	5.4 (±1.3)	0.04

**Table 4:** Correlation between diaphragmatic parameters, gestational age and birth weight. DTi: Diaphragmatic Inspiratory thickness; DTe: Diaphragmatic Expiratory thickness; DTf: Diaphragm thickening fraction; DE: Diaphragmatic excursion.

	Diaphragmatic Parameters	Correlation Coefficient r	*p*-Value
**Gestational age**	DTi	0.49	0.01
	DTe	0.52	0.01
	DE	0.24	0.13
	DTf	−0.19	0.23
**Birth weight**	DTi	0.47	0.02
	DTe	0.45	0.04
	DE	0.27	0.09
	DTf	−0.07	0.68

**Table 5:** Agreement between diaphragmatic measurements performed by operator 1 and operator 2. DTi: Diaphragmatic Inspiratory thickness; DTe: Diaphragmatic Expiratory thickness; DE: Diaphragmatic excursion.

Diaphragmatic Parameters	Operator 1	Operator 2	Difference	Limits of Agreement	ICC	CV, %
**DTi**	2.6± 0.6	2.7 ± 0.5	0.2 ± 0.8	−1.2–0.8	0.52	21.1%
**DTe**	2.3 ± 0.6	2.4 ± 0.5	0.2 ± 0.8	−1.0–0.7	0.66	22.8%
**DE**	4.9 ± 1	5.2 ± 1.2	0.3± 1.5	−2.7–2.2	0.32	21.7%

## Data Availability

The data presented in this study are available on request from the corresponding author.

## Supplementary Materials

The following supporting information can be downloaded at: https://www.mdpi.com/article/10.3390/children13010024/s1, Table S1: Shapiro–Wilk normality test for all continuous variables included in the analysis. DTi: Diaphragmatic Inspiratory thickness; DTe: Diaphragmatic Expiratory thickness; DTf: Diaphragm thickening fraction; DE: Diaphragmatic excursion.; Table S2: Correlation between diaphragmatic excursion at T1 and Silverman score, LUS score and the hours of ventilation. DE: Diaphragmatic excursion, LUS: lung ultrasound score.; Figure S1: Bland–Altman analysis of inter-operator agreement for diaphragmatic excursion (DE) measurements. The solid line indicates the mean bias, and dashed lines represent the 95% limits of agreement.; Figure S2. Bland–Altman analysis of inter-operator agreement for end-inspiratory diaphragmatic thickness (DTi) measurements. The solid line indicates the mean bias, and dashed lines represent the 95% limits of agreement.; Figure S3. Bland–Altman analysis of inter-operator agreement for end-expiratory diaphragmatic thickness (DTe) measurements. The solid line indicates the mean bias, and dashed lines represent the 95% limits of agreement.

## Abbreviations

The following abbreviations are used in this manuscript:

TTN	transient tachypnea of the newborn
NIV	non-invasive ventilation
GA	gestational age
BW	birth weight
DTi	End-inspiratory diaphragmatic thickness
DTe	End-expiratory diaphragmatic thickness
DE	Diaphragmatic excursion
DTf	Diaphragm thickening fraction
nCPAP	nasal Continuous Positive Airway Pressure
HHHFNC	Heated Humidified High-Flow Nasal Cannula
RDS	respiratory distress syndrome
LUS	Lung Ultrasound Score
SD	Standard deviation
IQR	interquartile range
CV	coefficient of variation
ICC	intraclass correlation coefficient
BPD	bronchopulmonary dysplasia
COPD	chronic obstructive pulmonary disease
