# Supplementary material for: Diaphragmatic Ultrasound in Neonates with Transient Tachypnea: Comparison with Healthy Controls and Inter-Operator Reliability
Source: Children (Basel). 2025 Dec 23;13(1):24. doi: 10.3390/children13010024 (PMC12839859; doi:10.3390/children13010024)
Supplement: Supplementary file 1 [file children-13-00024-s001.zip › children-3988760-supplementary.pdf]

Supplement Tables and Figures

	Shapiro-Wilk p value
**DTi T0**	0,7
**DTe T0**	0,4
**DTf T0**	0,1
**DE T0**	0,4
**DTi T1**	0,4
**DTe T1**	0,7
**DTf T1**	0,05
**DE T1**	0,06

Supplement Table S1: Shapiro–Wilk normality test for all continuous variables included in the analysis. DTi: Diaphragmatic Inspiratory thickness; DTe: Diaphragmatic Expiratory thickness; DTf: Diaphragm thickening fraction; DE: Diaphragmatic excursion.

	Correlation coefficient r	p value
**DE T1 and Silverman score T1**	-0,26	0,27
**DE T1 and LUS T1**	-0,44	**0,05**
**DE T1 and hours of ventilation**	0,35	0,13

Supplement Table S2: Correlation between diaphragmatic excursion at T1 and Silverman score, LUS score and the hours of ventilation. DE: Diaphragmatic excursion, LUS: lung ultrasound score.

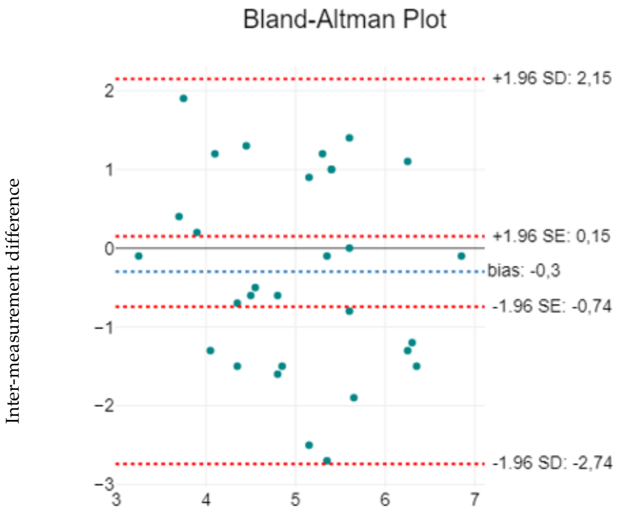

Mean of the two measurements

Supplement Figure S1. Bland–Altman analysis of inter-operator agreement for diaphragmatic excursion (DE) measurements. The solid line indicates the mean bias, and dashed lines represent the 95% limits of agreement.

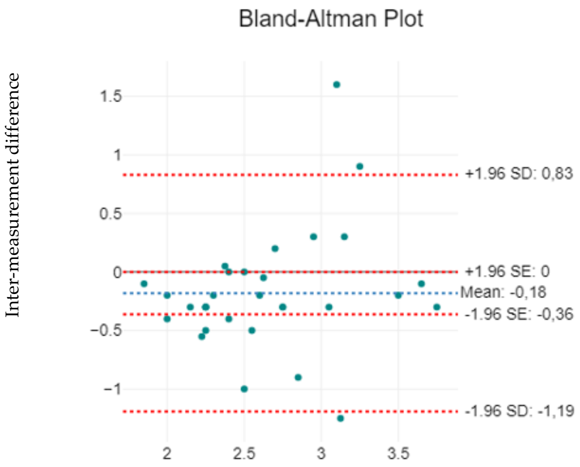

Mean of the two measurements

Supplement Figure S2. Bland–Altman analysis of inter-operator agreement for end-inspiratory diaphragmatic thickness (DTi) measurements. The solid line indicates the mean bias, and dashed lines represent the 95% limits of agreement.

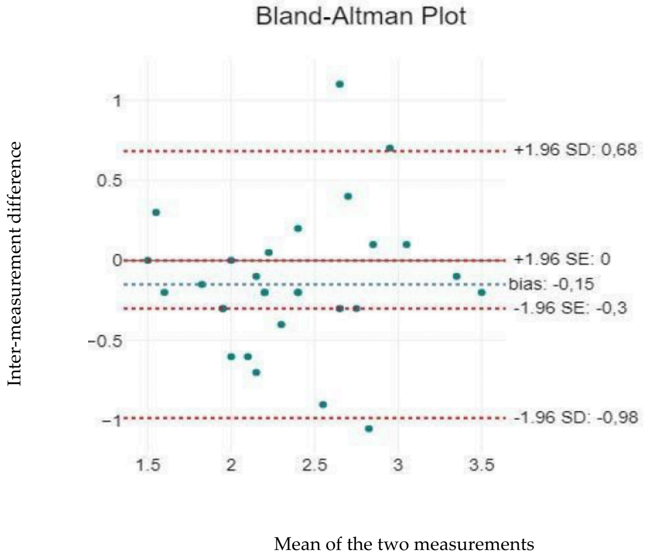

Supplement Figure S3. Bland–Altman analysis of inter-operator agreement for end-expiratory diaphragmatic thickness (DTe) measurements. The solid line indicates the mean bias, and dashed lines represent the 95% limits of agreement.
